# Determinants of stillbirth in Felege-Hiwot comprehensive specialized referral hospital, North-west, Ethiopia, 2019

**DOI:** 10.1186/s13104-019-4621-5

**Published:** 2019-09-14

**Authors:** Daniel Tarekegn Worede, Gizachew Worku Dagnew

**Affiliations:** 1 Department of Epidemiology and Biostatistics, School of Public Health, College of Medicine and Health Science, Bahir Dar University, Bahir Dar, Ethiopia; 2 Department of Reproductive Health, School of Public Health, College of Medicine and Health Science, Bahir Dar University, Bahir Dar, Ethiopia

**Keywords:** Stillbirth, Case–control, Determinants, Ethiopia

## Abstract

**Objective:**

The objective of this study was to identify determinants of stillbirth in Felege Hiwot comprehensive specialized referral hospital, North-west, Ethiopia: 2019. To conduct this study an institutional-based unmatched case–control study was used among 84 cases and 336 controls. Pretested, structured questioner with face to face interview was conducted and some data were also extracted from medical records using a checklist. The data were analyzed by using binary logistics regression. A p-value of < 0.05 was considered as significant at 95% confidence level and the strength of association was measured using odds ratio.

**Results:**

Illiteracy (AOR 3.8, 95% CI 1.4–10.2), sexually transmitted infection (AOR 5.7, 95% CI 1.1–29.7), Premature rupture of membrane (AOR 4.0, 95% CI 1.4–11.3), congenital anomaly (AOR 10.4, 95% CI 2.0–11.2) and history of perinatal death (AOR 10.4, 95% CI 3.7–29.2) were the determinants of stillbirth that increase risk of fetal death. Whereas taking at least two doses of tetanus toxoid vaccine (AOR 0.5, 95% CI 0.2–0.9) and partograph use (AOR 0.2, 95% CI 0.1–0.4) were found to be protective factors for stillbirth. To overcome this problem; empowering female education, facilitating women in taking tetanus toxoid vaccine, sexually transmitted infection prevention, and encourage health professionals to use partograph during labour follow up highly strongly recommended.

## Introduction

Stillbirth is defined as a fetus born with no sign of life, weighing more than 1000 g or with more than 28 completed weeks of gestation either as pre-partum or intrapartum stillbirth [1]. It is a neglected Tragedy with an estimated 2.6 million deaths per year globally and every day 7000 women experience a stillbirth worldwide [2]. All most all (99%) occur in low- and middle-income countries and 60% occur in rural areas. Half of these deaths occurred after labour had begun [2–4]. The mean stillbirth rate for Africans from 2010 to 2016 was 21.3 per 1000 births [4]. The 2016 Ethiopian demographic and health survey showed that the stillbirth rate was 11.8 per 1000 pregnancies at national and 50 deaths per 1000 pregnancies at Amhara region, where the study was conducted [5].

Stillbirths comprise a large proportion of preventable deaths, most of these losses are believed to be preventable with high-quality, evidence-based interventions delivered before and during pregnancy, labor, and delivery. Prevention of stillbirth was one of the new worldwide goals for maternal and child survival in millennium development goals, even though there were efforts to achieve this goal, the number of annual stillbirths remains unchanged since 2011 and is unacceptably high: an estimated 2.6 million in 2015. Indicators for stillbirth in post-2015 initiatives show that stillbirths are hidden in the worldwide agenda, and now continues in the sustainable development goal agenda [3]. Stillbirth rate as a health indicator plays an important role in providing the information needed to improve the health status of pregnant women and their newborns. Identifying determinants of stillbirth are very important for decision-makers in building and implement strategies to improve the care provided to pregnant mothers and their newborns.

According to some study findings; the burden of stillbirth was increased by mother with different comorbidities, preterm birth, and existing birth defect, on the other hand, uses of skilled maternal health services were the protective factors and can reduce it [3, 6–13]. But, advanced maternal age, maternal anemia, iron folate intake, TT vaccination, previous history of abortion, partograph use during labour follow up were not studied and documented in the study area in the best of authors’ knowledge. Therefore, this perspective unmatched case–control study was conducted in Felege Hiwot comprehensive specialized referral hospital to identify the determinants of stillbirth among mothers who had given birth in the study period from January 1 to 30th April 2019, North-west, Ethiopia.

## Main text

### Study setting and participants

Institutional based unmatched case–control study was conducted in Felege Hiwot comprehensive specialized referral hospital from 1st January to 30th April 2019. All still and live births after 28 weeks of pregnancy in the study period were considered as source populations. Still, and live births after 28 weeks of gestation in the study period were eligible for cases and controls, respectively while live and stillbirths with maternal mortality were excluded in the study.

The sample size was calculated based on an unmatched case–control formula by using Epi-info version-7 with the assumption of power = 95%, two-sided level significance = 95%, 1:4 case to control ratio. From previous case–control studies on determinants of stillbirth, one of the determinants was women’s with high hemoglobin levels (controls exposed = 6%, AOR = 4.64), then add 15% of nonresponse rate, and the final sample size was 420 (84 cases and 336 controls); which was the largest sample size from the alternative significant factors [12].

Data were collected in the labour wards by face to face interview using a pretested, structured questionnaire and checklist after identifying stillborn before the case mothers were discharged within a minimum of 12 h and 24 h for spontaneous and cesarean delivered mothers, respectively by BSC holder midwives who were worked in the Hospital. All mothers who had encountered stillbirth were recruited as cases consecutively until the required sample size was reached and four controls per case were selected using a systematic sampling technique.

The dependent variable was stillbirth (1 = died and 0 = alive) whereas independent variables were maternal age, occupation, educational status, marital status, fetal sex, gravidity, birth interval, ANC visit, TT vaccination, FP use, Iron-folate intake, history of perinatal death, history of abortion, APH, PROM, obstructed labour, prolonged labour, hypertensive disorder, HIV, DM, anemia, STI, HBV infection, partograph use, qualification of birth attendant, smoking, chewing, drug use, alcohol consumption.

Case: is defined as fetal death after 28 weeks of pregnancy (either pre-partum or intrapartum stillbirth) and Control: is defined as live births after 28 weeks of pregnancy. Gestational age was determined using the obstetric ultrasound report.

The data quality was assured by translating the questionnaire to Amharic, the working language in the study area, and then translated back into English to check the consistency. Two days of training was given for data collectors. The overall activity was supervised regularly by gynecologist doctor. All completed questionnaire and checklist were examined for completeness and consistency during data management, storage, and analysis.

Some data collected through interviews were repeated in the data extraction checklist to cross-check data accuracy. For those noted discrepancies, the data from the medical record were taken. Data were coded, entered and cleaned using epi-info version 7 then analysis was performed using SPSS version 23. Descriptive statistics, bivariate and multivariate logistic regression analysis was done. The multivariate logistic regression model was fitted using a backward elimination technique. The fitness of the model was checked using a Hosmer–Lemeshow test of significance p-value was > 0.05. Odds ratio with 95% confidence interval was used to measure the strength of association, those variables p-value less than 0.05 were taken as significant.

### Results

A total of 420 (84 cases and 336 controls were) were interviewed with a response rate of 100%. The mean age was 26.4 ± 7.50 (SD) and 26.2 ± 2.88 years’ standard deviation (SD) for the case and control mothers’ respectively. About 20 (23.8%) of the case and 17 (%5.1) of control mothers were found blew 20 years old whereas 14 (16.7%) of the case and 13 (3.8%) of control mothers were found ≥ 35 years old. With regard to maternal educational level, 10 (11.9%) and 95 (28.3%) of case and control mothers had attended tertiary (college certificate and above) (Table 1).

**Table 1:** Socio-demographic and obstetric characteristic of cases and controls in Felege Hiwot comprehensive specialized referral hospital, North-west, Ethiopia, 2019

Variable category	Stillbirth	
	Case (N, %)	Control (N, %)
Maternal age in years		
< 20	20 (23.8)	17 (5.1)
20–34	50 (59.5)	306 (91.1)
≥ 35	14 (16.7)	13 (3.8)
Maternal educational status		
Not educated	39 (46.4)	54 (16.1)
Primary	16 (19.1)	93 (27.7)
Secondary	19 (22.6)	94 (27.9)
Tertiary	10 (11.9)	95 (28.3)
Residence		
Urban	41 (48.8)	231 (68.8)
Rural	43 (51.2)	105 (31.2)
Sex of the neonate		
Female	49 (58.3)	193 (57.4)
Male	35 (41.7)	143 (42.6)
Number of fetus born		
Single	81 (96.4)	329 (97.9)
Twin and above	3 (3.6)	7 (2.1)
Four ANC visit		
No	37 (44.0)	52 (15.5)
Yes	47 (56.0)	284 (84.5)
At least two doses of TT vaccination		
Not received	42 (50.0)	51 (15.2)
Received	42 (50.0)	285 (84.8)
Modern family planning use before index pregnancy		
No	39 (46.4)	75 (22.3)
Yes	45 (53.6)	261 (77.7)
Iron-folate intake during pregnancy		
No	55 (65.5)	120 (35.7)
Yes	29 (34.5)	216 (64.3)
Previous history of abortion		
No	76 (90.5)	334 (99.4)
Yes	8 (9.5)	2 (0.6)
Previous history of perinatal death		
No	77 (91.7)	329 (97.9)
Yes	7 (8.3)	7 (2.1)
Birth interval for index pregnancy (months)		
< 24	49 (58.3)	98 (29.2)
≥ 24	35 (41.7)	238 (70.8)

From obstetric factors: mothers who had attended four and above antenatal care follow up were 47 (56.0%) case mothers and 284 (84.5%) of control mothers, whereas 42 (50.0%) and 285 (84.8%) of case and control mothers had received at least two doses of tetanus toxoid vaccination during pregnancy. Twenty-nine (34.55) case mothers and 216 (64.3%) control mothers had taken an iron-folate pill during pregnancy. About, 8 (9.5%) of case mothers and 2 (0.6%) of control mothers, 7 (8.3%) of case mothers and 7 (2.1%) of control mothers had a history of abortion and perinatal deaths, respectively (Table 1).

From maternal and fetal related complications, and health care factors: about, 7 (8.3%) of case mothers and 4 (1.2%) of control mothers had a history of sexually transmitted infection during the index pregnancy. About, 16 (20.0%) cases and 20 (6.0%) of control mothers had anemia, whereas, about, 32 (38.1%) and 18 (5.4%) of the fetus had congenital malformation from case and control mothers, respectively. With regard to health care factors about, half (50.0%) the case mother labour and the majority 279 (83.0%) control mothers’ labour had followed using Partograph (Table 2).

**Table 2:** Maternal and fetal complications and health care characteristic of cases and controls in Felege-Hiwot comprehensive specialized referral hospital, North-west, Ethiopia, 2019

Variable category	Stillbirth	
	Case (N, %)	Control (N, %)
HIV positive		
No	79 (94.0)	334 (99.4)
Yes	5 (6.0)	2 (0.6)
PROM		
No	66 (78.6)	323 (96.1)
Yes	18 (21.4)	13 (3.9)
STI		
No	77 (91.7)	332 (98.8)
Yes	7 (8.3)	4 (1.2)
Anemia		
No	68 (81.0)	316 (94.0)
Yes	16 (20.0)	20 (6.0)
Congenital malformation		
No	52 (61.9)	318 (94.6)
Yes	32 (38.1)	18 (5.4)
Meconium aspiration syndrome		
No	63 (75.0)	327 (97.3)
Yes	21 (25.0)	9 (2.7)
Chewing Khat		
No	77 (91.7)	327 (97.3)
Yes	7 (8.3)	9 (2.7)
Partograph use during follow up		
No	42 (50.0)	57 (17.0)
Yes	42 (50.0)	279 (83.0)

#### Factors associated with stillbirth

In multivariable binary logistic regression analysis; women who were illiterate (AOR = 3.8, 95% CI 1.4–10.2) had high odds of stillbirth compared to women had tertiary education, women who had had sexually transmitted infection (AOR = 5.7, 95% CI 1.1–29.7), congenital anomaly (AOR = 10.4, 95% CI 2.0–11.2), premature rupture of membrane (AOR = 4.0, 95% CI 1.4–11.3), previous history of perinatal death (AOR = 10.4, 95% CI 3.7–29.2) had high odds of stillbirth compared to their counterparts; on the other hand, women taking at least two doses of tetanus toxoid vaccine during pregnancy (AOR = 0.5, 95% CI 0.2–0.9), and partograph use during delivery (AOR = 0.2, 95% CI 0.1–0.4) had low odds of facing stillbirth compared to their counterparts (Table 3).

**Table 3:** The determinants of stillbirth in Felege-Hiwot comprehensive specialized referral hospital, North-west, Ethiopia, 2019

Variable category	Stillbirth		COR 95% CI	AOR 95% CI
	Case (N, %)	Control (N, %)		
Educational status of the mother				
Not educated	39 (46.4)	54 (16.1)	6.9 (3.2–14.8)	3.8 (1.4–10.2)
Primary	16 (19.1)	93 ( 27.7)	1.6 (0.7–3.8)	1.6 (0.6–4.4)*
Secondary	19 (22.6)	94 (27.9)	1.9 (0.8–4.3)	1.9 (0.8–5.3)*
Tertiary	10 (11.9)	95 (28.3)	1	1
At least two doses of TT vaccine				
Yes	41 (48.8)	231 (68.8)	0.2 (0.1–0.3)	0.5 (0.24–0.9)
No	43 (51.2)	105 (31.2)	1	1
History of abortion				
No	49 (58.3)	193 (57.4)	1	1
Yes	35 (41.7)	143 (42.6)	17.6 (3.7–84.4)	6.33 (0.8–47.6)*
PROM				
No	81 (96.4)	329 (97.9)	1	1
Yes	3 (3.6)	7 (2.1)	6.8 (3.2–14.5)	4.03 (1.4–11.3)
STI				
No	77 (91.7)	332 (98.8)	1	1
Yes	7 (8.3)	4 (1.2)	7.5 (2.2–26.4)	5.74 (1.1–29.7)
Congenital anomaly				
No	52 (61.9)	318 (94.6)	1	1
Yes	32 (38.1)	18 (5.4)	10.9 (5.7–20.8)	4.78 (2.0–11.2)
History of perinatal death				
No	77 (91.7)	329 (97.9)	1	1
Yes	7 (8.3)	7 (2.1)	12.1 (5.3–27.7)	10.4 (3.7–29.2)
Partograph use				
No	42 (50.0)	57 (17.0)	1	1
Yes	42 (50.0)	279 (83.0)	0.20 (0.12–0.34)	0.2 (0.1–0.4)

* Non-significant; 1 = reference category

### Discussion

This study investigated the determinants of stillbirth at Felege-Hiwot comprehensive specialized referral hospital in North-west, Ethiopia.

The odds of experiencing stillbirth were 3.8 fold higher among mothers who had no education compared to those who had attended tertiary and above education level. The finding was consistent with the study [6] conducted in the Amhara region, Nigeria [14] and the other study conducted in low resource settings [15]. This might be due to illiteracy that might compromise economic status, access to health care and birth spacing. The other reason might be the knowledge difference among mothers for skilled maternal health services use.

The odds of experiencing stillbirth were 5.7 times more likely among mothers who had a history of sexually transmitted infections during pregnancy compared to their counterparts. The finding of the current study was consistent with the study conducted in Brazil [16]. This might be due to infection transmission via the placenta, which causes fetal infection and might leads to pregnancy loss.

Premature rupture of the membrane was one of the strongest predictors of stillbirth in the study, mothers who had encountered premature rapture of membrane had fourfold odds of stillbirth than mothers who had no premature rupture of membrane. The result of the study was in line with the studies conducted in Nigeria [14] and low resource settings [17]. The possible explanation might be fetus might have encountered perinatal asphyxia, which causes fetal death.

The study found that congenital anomaly was significantly associated with stillbirth. The odds of experiencing stillbirth were higher among fetal deaths born with congenital anomaly compared with a fetus with no congenital defects. This finding was supported by the study conducted in Nigeria [14]. The possible reason for this might be if there is a congenital anomaly the fetus might have organ dysfunction, which leads to fetal complication and death.

The other determinant factors for still-birth were a history of perinatal death. The odds of experiencing stillbirth were higher among women who had a history of perinatal loss compared to their counterparts. This finding was in line with the study conducted in Nepal [10]. The first treason for this might be related to the maternal Rh-factor, which leads to erythroblastosis fetalis. The second reason might be maternal chronic and repeated pregnancy-related comorbidities that result in fetal deaths.

Taking at least two doses of tetanus toxoid vaccine during pregnancy was a protective factor associated with decreasing the risk of stillbirth. The odds of experiencing stillbirth were 50% less likely among women who had taken at least two doses of tetanus toxoid (TT) vaccine during pregnancy compared to those women who had no received two doses of the tetanus toxoid vaccine during pregnancy. The possible explanation might be if the mother had taken at least two doses during or before conception, the fetus became protected from tetanus in the womb, and this might protect the fetus from birth defects, miscarriage and stillbirth.

The other protective determinant factors associated with decreasing the risk of stillbirth were partograph use during labour follow up. The risk of fetal loss was decreased by 80% for those mothers whose labour had monitored by using partograph compared who didn’t. The reason for this might be due to using a partograph that may be helpful in early detection of fetal and maternal complications, and this can help the gynecologist to take action to save the life of the fetus and mothers if there is a sign of feto-maternal complication.

The determinants of stillbirth in the current study were; easily identifiable and manageable with existing basic obstetric and neonatal care. While empowering female education, facilitating women in taking tetanus toxoid vaccine before and during pregnancy, STI prevention and treatment, and encouraging health professionals to use partograph during labour follow up could reduce the burden of stillbirth.

## Limitation of the study

The health institution based study might have had an over-representation of the determinants of stillbirth, as more complicated cases are referred and since the data was collected in the institution only the stillbirths in the community were no considered. The homogeneity of the study population might not be considerable compared to other health facilities since it was conducted only one largest regional comprehensive specialized referral hospital.

## Data Availability

The data is available in SPSS.

## Abbreviations

ANC: antenatal care
APH: ante-partum hemorrhage
BDU: Bahir Dar University
BSC: Bachelor of Science
FP: family planning
HBV: hepatitis-b virus
PROM: premature rupture of membrane
STI: sexual transmitted infection
TT: tetanus toxoid
